# Postoperative Vision Loss after Reverse Shoulder Arthroplasty

**DOI:** 10.1155/2014/850950

**Published:** 2014-12-24

**Authors:** Aadil Mumith, John Scadden

**Affiliations:** ^1^Royal National Orthopaedic Hospital, Brockley Hill, Stanmore HA7 4LP, UK; ^2^Trauma & Orthopaedic Department, St. Mary's Hospital, Parkhurst Road, Newport, Isle of Wight PO30 5TG, UK

## Abstract

We report a case which highlights the rare but devastating complication of postoperative vision loss (POVL) in orthopaedic surgery. Though documented previously, it has not been reported in shoulder arthroplasty surgery of which we present the first case. The aetiology of POVL is difficult to elucidate due to its elusive nature. We explain the risks associated with regional blocks used for such surgery and how this may be related to POVL. We must be vigilant of the possible causes of POVL as curative treatment is often not possible and hence must take preventative measures which we have recommended. Fortunately, the patient fully recovered at 10 months postoperatively with excellent function of her reverse shoulder arthroplasty.

## 1. Introduction

Postoperative vision loss (POVL) is a rare but documented phenomenon in nonocular surgery. To our knowledge, only 2 previous cases [1–3] reported POVL in patients undergoing shoulder surgery. We present the first reported case of POVL in a patient undergoing reverse shoulder arthroplasty (RSA). The elusive nature of POVL makes it very difficult to elucidate its aetiology as incidence rates in nonocular surgery range from 0.013% to 0.2% [4].

Loss of upper limb function can impair activities of daily living as well as employment status, recreational activity, and psychological health [5].

Nonunions of conservatively managed proximal humeral fractures are usually related to old age, metaphyseal comminution, and fracture displacement [6]. There are a number of surgical options in managing proximal humeral nonunion including reverse-geometry shoulder arthroplasty (RSA).

RSA is becoming more common in practice but is not without its risks. The complication rates for RSA were high when these were first introduced, but with advancing implant design and surgical technique the majority of RSA specific complications have been minimised with only a handful of complications which still need to be addressed which include glenoid loosening, acromial fractures, dislocation, infection, nerve palsy, and notching. Neurological complications include clinical palsies of the musculocutaneous nerve in up to 1.4% of patients and subclinical neurological disturbance predominantly involving the axillary nerve in up to 45% of patients detected on electromyography [7]. To our knowledge, there have been no reported cases involving any neurological disturbance leading to visual loss.

Curative treatment is often not possible if POVL has occurred and therefore high vigilance and preventative measures must be undertaken to avoid this devastating complication. This case highlights a potential disastrous complication following shoulder arthroplasty of which to be aware.

## 2. Case Report

**Figure 1:**
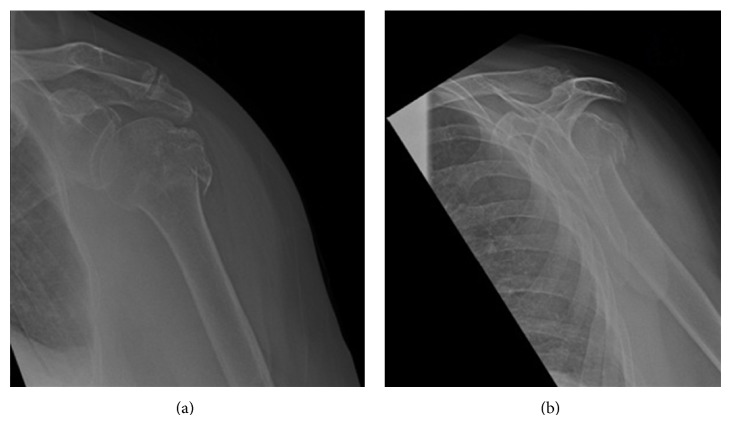
AP (a) and lateral (b) radiographs of left proximal humeral fracture.

A 76-year-old independent lady sustained a low energy mechanical fall. She presented to the emergency department with a closed, neurovascularly intact isolated injury to her left shoulder. Radiographs confirm a minimally displaced 3-part fracture of her left proximal humerus (Figure 1).

She has a background of polymyalgia rheumatica (PMR), hypertension, hypercholesterolemia, osteoporosis, rheumatoid arthritis, recurrent anterior uveitis, and early bilateral cataracts for which she is being followed up by ophthalmology with no worsening in her symptoms. Her regular medications include alendronic acid, calcium supplements, aspirin, hypromellose, Maxidex, omeprazole, paracetamol, prednisolone, simvastatin, and tramadol. She is a nonsmoker.

**Figure 2:**
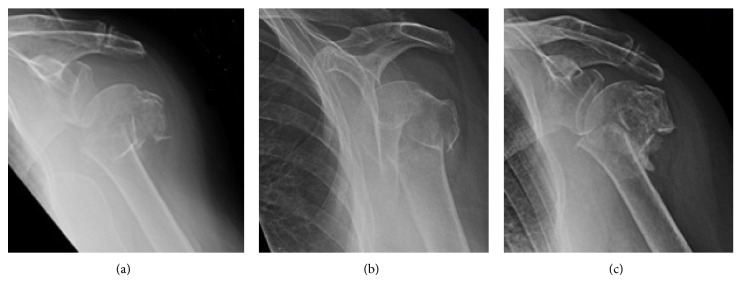
AP radiographs of left proximal humerus showing initial displacement (a) and reduction with conservative management (b), leading to painful nonunion at 4-month stage.

Her arm was immobilised in a collar and cuff and it was decided to manage her conservatively with routine initial weekly follow-up in outpatients. At two weeks, her fracture was displaced further but given her pain-free range of movement it was decided to continue conservative management. She was seen regularly over a number of weeks in the fracture clinic with a satisfactory comfortable range of movement and an adequately reduced fracture radiographically. At 4 months, however, she presented with a very painful shoulder which she could hardly move. Radiographs confirmed nonunion and it was decided to proceed with a reverse-geometry shoulder replacement under general anaesthesia supplemented with an interscalene block (ISB) (Figure 2).

On the day of the procedure, she underwent standard induction with dexamethasone 4 mg, ondansetron 4 mg, propofol 20 mg, fentanyl 100 mg, and rocuronium 50 mg. Sevoflurane was used as an anaesthetic agent. The ISB was ultrasound-guided and furthermore aided with a nerve stimulator; all of them were carried out under sterile technique with 20 mL 0.5% chirocaine (levobupivacaine hydrochloride) infiltrated. She had standard SpO_2_, blood pressure, ECG, and ETCO_2_ and anaesthetic agent monitoring throughout the procedure. Antibiotic prophylaxis was given during induction in keeping with departmental protocols.

**Figure 3:**
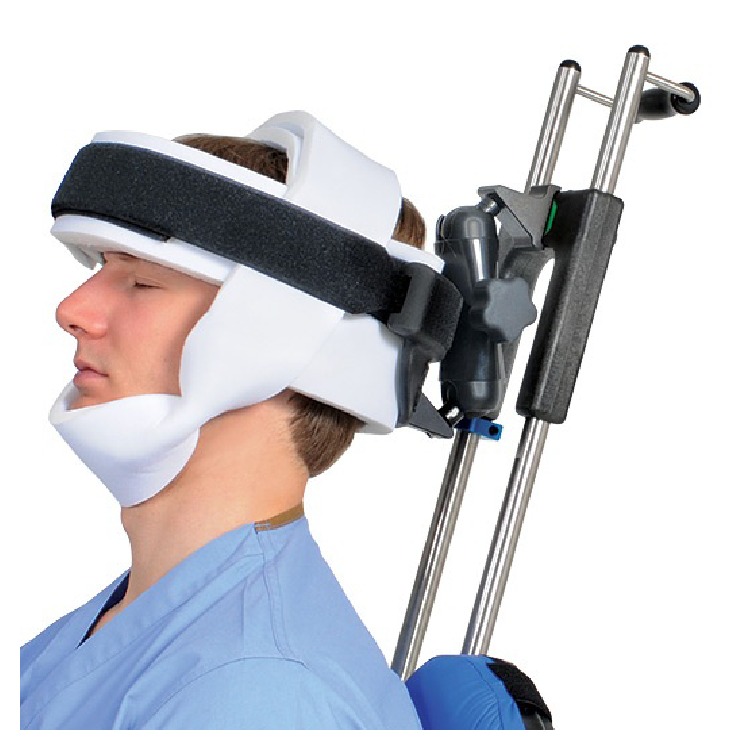
Picture of Allen Universal Head Restraint with Allen Universal Head Positioner taken from http://www.allenmedical.com.

She was placed in the beach-chair position [8] with use of the Allen Lift-Assist Beach Chair (Allen Medical, MA, USA). Her head was secured with the Allen Universal Head Positioner together with the Allen Universal Head Restraint (Figure 3). This comprises a foam strap that is wrapped around the forehead of the patient together with a chin strap; both are Velcro fastened. Her eyes were covered with light cotton wool pads secured with micropore (3M, MN, USA) tape.

A standard deltopectoral approach was used and a SMR (Lima Corporate SPA, Udine, Italy) reverse-geometry shoulder replacement was implanted using the surgeon's default technique. Intraoperatively, she was found to have very osteoporotic bone and sustained a small glenoid rim fracture whilst applying the baseplate; this was replaced with a larger baseplate that was secure. There were no other issues surgically or anaesthetically with her systolic blood pressure remaining above 90 mmHg at all times. The operation lasted approximately 2.5 hours.

Upon waking from the anaesthesia in the recovery room, much to the surprise of the anaesthetic and surgical teams, the patient reported reduced vision in her left eye. Ophthalmology review revealed that her visual acuity had declined to only hand movements with an associated relative afferent papillary defect. Fundoscopy showed chronic changes but was otherwise unremarkable and an urgent CT head excluded any intracranial pathology. The following day, her vision had improved slightly but the relative afferent papillary defect remained together with an associated total loss of colour vision with some mild retinal pallor seen on fundoscopy. It was concluded by the ophthalmology team that she had most likely suffered from a central retinal artery occlusion (CRAO) with some underperfusion of the retina as a result. Bilateral carotid Doppler revealed atherosclerotic disease suggested by presence of intima thickening and smooth noncalcified plaques. Her visual acuity slowly improved; however, she had not recovered her colour vision at one month postoperatively. Ophthalmologists proceeded with temporal artery biopsies to aid in excluding temporal arteritis but histopathology was inconclusive. Two months postoperatively, her vision had almost improved completely (left eye 6/9); however it declined dramatically at 6 months (left eye 6/60) due to progression of her cataracts for which she underwent surgery. 10 months from her original shoulder operation, her vision was completely normal (left eye 6/6).

**Figure 4:**
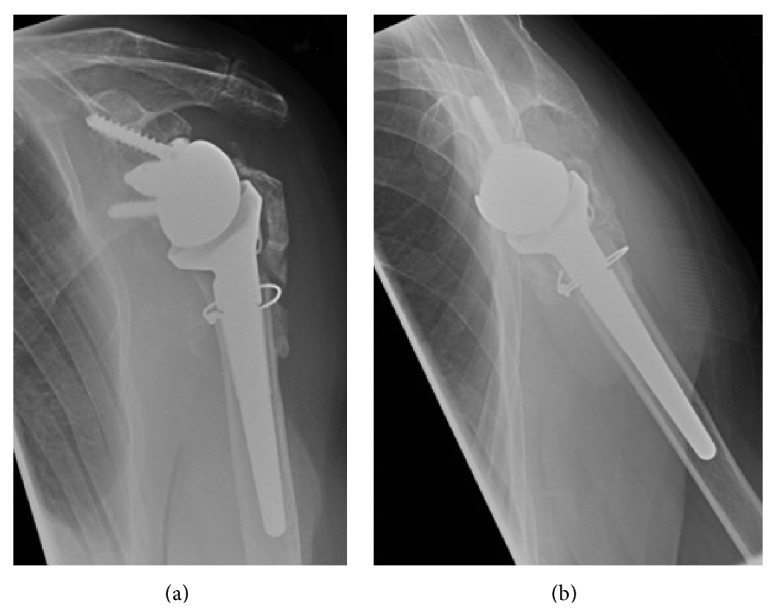
Final AP (a) and lateral (b) radiographs of RSA at 6 months. Default surgical technique is to place wire around proximal humerus to prevent its fracturing during preparation and press fit of humeral component.

Her standard orthopaedic follow-up for her reverse-geometry shoulder replacement was unremarkable with an excellent range of movement achieved at 8 weeks with satisfactory radiographs (Figure 4) and she was discharged at the 6-month stage.

## 3. Discussion

Postoperative vision loss (POVL) in patients undergoing nonocular surgery under general anaesthesia is caused by anterior or posterior ischaemic optic neuropathy (AION/PION), central retinal artery occlusion (CRAO), pituitary apoplexy, or cortical blindness [9].

Several retrospective studies have identified the incidence of POVL to range from 0.0009% to 16.3% with spinal surgery and surgery involving cardiopulmonary bypass being more commonly associated [1].

As of 2010, 93 spinal surgical cases of POVL were submitted to the American Society of Anaesthesiologists POVL Registry, with only 10 of them attributed to CRAO. CRAO has been well documented in the literature being caused by direct ocular trauma [10], thromboembolic events [11], or vasospastic episodes [12]. The majority of those patients experiencing POVL occur in cases where the patient is positioned prone or if there is prolonged excessive ocular pressure secondary to the anaesthetic mask in those patients positioned supine [13]. We feel that it is very unlikely for the POVL experienced in our case to be due to external ocular pressure. The senior author is very experienced with the use of the head positioning system and it had been used as standard in our unit for over a year for all types of shoulder surgery. As the drapes were removed postoperatively, the head restraint had not migrated and was as at the start of the procedure. Intraoperatively, the anaesthetic team had checked the position of the patient's head throughout the procedure.

POVL may be attributed to thromboembolic phenomena. Our patient had a background of hypertension and hypercholesterolemia, prime risk factors for atherosclerosis. It is a reasonable presumption that the patient, over the years, may have developed atherosclerotic plaques within her arteries. The terminal event of plaque development is rupture. This can cause occlusion either at the site of the rupture via endothelial cellular events or distally as a result of plaque embolisation [14]. Smooth noncalcified plaques were confirmed on carotid Doppler; it is therefore logical to suggest that her CRAO may have been a result of a plaque embolism through her arterial circulation, more commonly known as amaurosis fugax. This could be just an unfortunate set of circumstances where the patient had spontaneous fat emboli intraoperatively or possibly secondary to arterial vasospasm dislodging some of the plaque.

Vasospasm can be caused either by direct trauma in the form of arterial puncture or chemically due to the local anaesthetic infiltration [15]. We accept that it is unlikely that the needle from the ISB will cause direct trauma to the carotid artery as ultrasound was used to aid the procedure; however it still remains a possibility and is a recognised risk. The vertebral artery is also a potential site at risk of mechanical vasospasm during ISB due to its proximity to route of the needle [16]. Theoretically chemically induced vasoconstriction can be caused by the levobupivacaine used for the ISB. At clinical doses, aminoamides are well known to have vasodilatory effects but at subclinical doses it has been shown that levobupivacaine is the most potent vasoconstrictor when compared to other aminoamides [17].

We note that the patient had an existing ophthalmological history of recurrent anterior uveitis and bilateral cataracts. These conditions were stable at the time of surgery and there is no literature to support an increased risk of POVL in nonocular surgery in those patients with preexisting eye pathologies.

## 4. Conclusion

As orthopaedic surgeons, a major part of our duty of care is to provide the safest possible service to our patients. It is very difficult to delineate the cause of POVL in patients in nonocular surgery given its rarity and even less so in those patients undergoing upper limb orthopaedic procedures.

In order to decrease the likelihood of POVL, we recommend the ISB to be carried out under ultrasound guidance so that a higher concentration of a smaller volume of local anaesthetic can be administered accurately. Adequate but not excessive padding of the eyes to prevent unnecessary extraocular pressure must be placed. A safe and secure head support is also advised. Intraoperative care must be taken so as not to lean on the padding securing the patients head or to that covering the eyes. Postoperatively, the patients' vision (gross visual fields not fundoscopy) must be checked by either the anaesthetist or the recovery room staff to identify any visual problems early.

This case highlights a devastating complication of upper limb surgery and is the first case of POVL to be reported in shoulder arthroplasty surgery. We feel that being aware of the possible causes and adhering to the recommendations made, although the exact aetiology of POVL is unknown, can minimise the risk.

## References

[1] Bhatti M. T., Kayser Enneking F. (2003). Visual loss and ophthalmoplegia after shoulder surgery. *Anesthesia and Analgesia*.

[2] Gilbert M. E., Savino P. J., Sergott R. C. (2006). Anterior ischaemic optic neuropathy after rotator cuff surgery. *British Journal of Ophthalmology*.

[3] Fournier J. H., Velis E. (2004). Visual loss after shoulder surgery under general anesthesia diagnosed as caused by ocular compression with electro-retinography testing: case report and review of the literature. *International Surgery*.

[4] Berg K. T., Harrison A. R., Lee M. S. (2010). Perioperative visual loss in ocular and nonocular surgery. *Clinical Ophthalmology*.

[5] Datta D., Selvarajah K., Davey N. (2004). Functional outcome of patients with proximal upper limb deficiency—acquired and congenital. *Clinical Rehabilitation*.

[6] Court-Brown C. M., McQueen M. M. (2008). Nonunions of the proximal humerus: their prevalence and functional outcome. *The Journal of Trauma*.

[7] Smith C. D., Guyver P., Bunker T. D. (2012). Indications for reverse shoulder replacement. *The Journal of Bone and Joint Surgery B*.

[8] Peruto C. M., Ciccotti M. G., Cohen S. B. (2009). Shoulder arthroscopy positioning: lateral decubitus versus beach chair. *Arthroscopy—Journal of Arthroscopic and Related Surgery*.

[9] Dickemper R. L., Griffin A. T. (2010). Vision loss as a complication of nonophthalmologic surgery: implications for care for the perianesthesia nurse. *Journal of Perianesthesia Nursing*.

[10] Noble M. J., Alvarez E. V. (1987). Combined occlusion of the central retinal artery and central retinal vein following blunt ocular trauma: a case report. *British Journal of Ophthalmology*.

[11] Kollarits C. R., Lubow M., Hissong S. L. (1972). Retinal strokes. I. Incidence of carotid atheromata. *Journal of the American Medical Association*.

[12] Brown G. C., Magargal L. E., Shields J. A., Goldberg R. E., Walsh P. N. (1981). Retinal arterial obstruction in children and young adults. *Ophthalmology*.

[13] Grossman W., Ward W. T. (1993). Central retinal artery occlusion after scoliosis surgery with a horseshoe headrest: case report and literature review. *Spine*.

[14] Kolodgie F. D., Burke A. P., Farb A., Gold H. K., Yuan J., Narula J., Finn A. V., Virmani R. (2001). The thin-cap fibroatheroma: a type of vulnerable plaque: the major precursor lesion to acute coronary syndromes. *Current Opinion in Cardiology*.

[15] Johns R. A., DiFazio C. A., Longnecker D. E. (1985). Lidocaine constricts or dilates rat arterioles in a dose-dependent manner. *Anesthesiology*.

[16] Carden E., Ori A. (2005). Applying cervical spine anatomy to interscalene brachial plexus blocks. *Pain Physician*.

[17] Sung H.-J., Ok S.-H., Sohn J.-Y., Son Y. H., Kim J. K., Lee S. H., Han J. Y., Lim D. H., Shin I.-W., Lee H.-K., Chung Y.-K., Choi M.-J., Sohn J.-T. (2012). Vasoconstriction potency induced by aminoamide local anesthetics correlates with lipid solubility. *Journal of Biomedicine and Biotechnology*.

